# Ulcus Vulvae Acutum Lipschütz—UVAL: case series at a Swiss university hospital emergency room

**DOI:** 10.3389/frph.2023.1333620

**Published:** 2024-01-16

**Authors:** Vittoria Guareschi, Patrice Mathevet, Martine Jacot-Guillarmod

**Affiliations:** ^1^ Faculty of Biology and Medicine (FBM), University of Lausanne, Lausanne, Switzerland; ^2^ Department of Obstetrics and Gynecology, Lausanne University Hospital, University of Lausanne, Lausanne, Switzerland

**Keywords:** Ulcus Vulvae Acutum Lipschütz, diagnostic algorithm, gynecological emergency room, flu-like symptoms, HSV1/2, EBV, Behcet disease

## Abstract

**Background:**

Ulcus Vulvae Acutum Lipschütz (UVAL) is a largely unknown disease with a broad and complex differential diagnosis.

**Objectives:**

To provide a description of the main characteristics of UVAL, determine the most appropriate diagnostic process and describe the current therapeutic approach.

**Methods:**

We designed a retrospective, descriptive cohort study using the gynecological-ER database of our institution. *Inclusion criteria:* female patients aged between 10 and 20 years old with suspicion of a UVAL diagnosis at CHUV's gynecological ER. *Data extraction*: epidemiological characteristics, clinical presentation, laboratory tests, established diagnostics, treatment, and ulcer outcomes.

**Results:**

15 patients were included for the analysis; average age: 15 years old; 60% of patients were virgo at the time of ulcer onset; all patients had at least one flu-like symptom concomitant with the vulvar lesion; the most-performed serology was for EBV and acute disease was present in only one patient; for diagnostic purposes two biopsies were performed with both inconclusive histopathology analysis; the main prescribed treatments were: oral NSAIDs, Paracetamol, and Lidocaine gel; 93% of cases presented signs of regression; the average follow-up time was 10 days. *The diagnostic algorithm of Sadoghi et al:* 10 out of 15 cases were retrospectively diagnosed with UVAL by the algorithm; half were diagnosed with UVAL, and the other half received a diagnosis of “ulcers of unknown origin” at the time of the gynecological ER visit.

**Conclusions:**

We highly recommend the diagnostic and therapeutic algorithms developed by Sadoghi et al. as valuable tools to guide clinical reasoning and, consequently, improve acute vulvar ulcers management.

## Introduction

First described by the Austrian dermatologist Benjamin Lipschütz (1), UVAL—Ulcus Vulvae Acutum Lipschütz—is a non-venereal disease affecting mainly, but not exclusively, young girls and adolescent (2, 3). More than 80 UVAL case reports are published in the literature and its incidence is estimated at 4%–35% of women presenting acute genital ulcers (4). It manifests itself with one or more highly painful vulvar ulcerations characterized by an acute onset. Their average duration is two weeks (5), and recurrences are possible but not frequent (6). Topical corticosteroids are frequently chosen for UVAL treatment. Nevertheless, spontaneous resolution of the ulcers is often described in existing literature (7–9). In a 60 cases meta-analysis, the proportion of UVAL patients reporting inaugural flu-like symptoms varies between 50% and 80% (8). In addition, the proportion of patients presenting themselves with systemic infection few days or weeks prior to ulcer onset (3, 9, 10) ranges between 30% and 80% (5, 11). An association with positive serology for viral and bacterial pathogens such as EBV, CMV, and Mycoplasma Pneumonia has been described in several case series and reports. Two hypotheses have been proposed: one suggests that the cytotoxic effect of the virus causes ulceration and triggers a febrile state in the patient, while the second hypothesis suggests that ulceration is a result of a non-specific inflammatory state in response to systemic viral infection. However, neither of these hypotheses has been histologically proven, and thus they are not considered sufficient to establish a diagnosis of UVAL (8). A venereal origin must be excluded: broaching this topic with young virgo patients may require sensitivity, especially when the adults responsible for their care are also involved in the process (12). Additionally, given their location and appearance, the possibility of a sexual abuse must also be questioned (7), making the context of medical history even more delicate. Systemic-non-infectious diseases such as Behçet's, Crohn's, autoimmune bullous diseases, complex aphthosis, or malignancies, may also manifest as acute vulvar ulceration (8), thus further complicating the diagnostic process (13, 14). Our case series aims to assist clinicians who will face UVAL disease within a complex setting such as pediatric and adolescent gynecological care.

## Methods

We conducted a retrospective, descriptive, cohort study, after receiving approval from Vaud's Ethics Committee (n° CER-VD 2021-00448) on 02/08/21. Inclusion criteria: patients aged 10–20 years old who consulted CHUV's gynecological ER between January 2000 and December 2020 for whom a diagnosis of UVAL could be suspected. Exclusion criteria: patients under 10 and over 20 years of age; patients who consulted outside the gynecological ER; patients without consent for the re-use of personal data for research purposes or whose request was pending. Additionally, for a more specific selection, we searched patients using five diagnostic codes of the International Classification of Diseases (15) and performed an in-depth keyword research (*acute vulvar ulcer, Lipschütz ulcer, vulvar ulcer, vulvar aphtha, Lipschütz, UVAL*). We excluded patients aged under 10 and over 20 years old due to the high UVAL incidence in this age range, in alignment with Sadoghi et al.'s findings. Managing sexual health issues in young patients can be challenging, especially within our gynecological-pediatric focused team, as it often involves delicate care associated with youth, sexual health, and the emotional strain it places on both healthcare providers and young patients and their families due to UVAL challenging diagnostic process.

### Data collection

**Figure 1:**
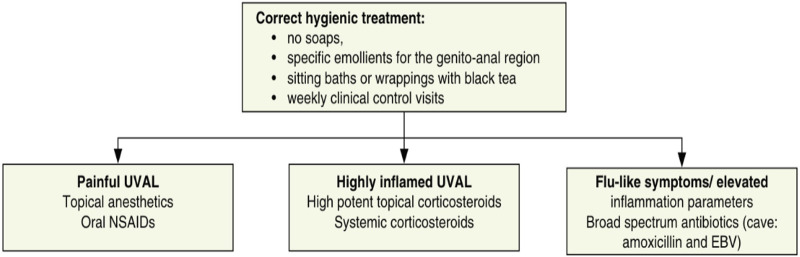
Therapeutic algorithm [Figures 2 of Sadoghi et al. (8) article].

We designed our study based on the 2020 paper by Sadoghi et al. (8), taking into consideration their diagnostic and therapeutic algorithms for UVAL management: Table 1 and Figure 1.

**Table 1:** Diagnostic algorithm [adapted from Sadoghi et al. (8) article].

Major criteria	Minor criteria
1. Acute onset of ≥1 painful ulcerous lesion in the vulvar region 2. Exclusion of infectious and other non-infectious causes for the ulcer (use Figures 3 and 4)	1. Localization of ulcer at vestibule or labia minora 2. No sexual intercourse ever (i.e., patient is a virgin) or within the last 3 months. 3. Flu-like symptoms 4. 4. Systemic infection within 2–4 weeks prior to onset of vulvar ulcer.

If both major criteria AND ≥2 minor criteria are present, then a diagnosis of UVAL is warranted.

We retrospectively applied this algorithm to our sample. To do so, we followed the diagnostic criteria listed therein and, for the sake of consistency, we extracted the same epidemiological and clinical data that Sadoghi et al. analyzed. Furthermore, inspired by their symptom-based therapeutic algorithm, we proceeded to classify the treatments prescribed in our case series.

Data was extracted by CHUV's data analyst via a coded, confidential process using the Didata system.

## Results

Specific diagnostic code and keyword research enabled us to identify a total of 42 patients suspect of UVAL diagnosis. Among these, we excluded 22 patients who sought consultation outside the gynecological emergency room: 10 patients in the pediatric department, 4 in the general emergency room, and the remaining 8 were in various departments such as immunology, dermatology, rheumatology, endocrinology, and obstetrics. We enrolled a total of 15 patients who met the inclusion criteria. The average age of the enrolled patients was 15 years old, with a range of 10–19 years.

*Epidemiological characteristics and medical history:* we classified our population into three groups: Group 1—patients who had engaged in sexual intercourse within the previous three months (27%); Group 2—non-virgo patients who were not sexually active within the three months before the ulcer lesion (13%); and Group 3—virgo patients (60%). Ten out of fifteen patients reported a history of canker sores, while five patients had previously experienced a similar episode of vulvar ulcer. At least one flu-like symptom was found in all patients; 73% presented with a febrile condition inaugurating the vulvar lesion and in 47% of cases the vulvar ulcer occurred in conjunction with upper respiratory tract infection symptoms. In the medical records of three patients, it was observed that clinicians initially considered the possibility of sexual abuse as the underlying cause of the vulvar lesion. However, these suspicions were eventually dismissed. Likewise, in two other cases, trauma (such as impact or blow, use of soaps or detergents, friction from tight underwear, etc…) was considered; nonetheless, this hypothesis was ultimately discarded as the final diagnostic conclusion (Table 2).

**Table 2:** Epidemiological characteristics and medical history.

	Average	Range	SD
Age (years)	15	10–19	2.6
Average BMI	19.1	15.2–22.8	2.5
Medical history	*n*	Percentage	
Gynecological history			
Group 1	4/15	27%	
Group 2	2/15	13%	
Group 3	9/15	60%	
Menstruated	12/15	80%	
Method of contraception used	5/15	33%	
• Pill	4/5	80%	
• Condom	1/5	20%	
History of STIs	1/15	7%	
History of genital ulcer	5/15	33%	
Flu-like symptoms (concomitant with vulvar ulcer)			
Febrile episode	11/15	73%	
RSV infection	7/15	47%	
Asthenia	5/15	33%	
Abdominal pain	5/15	33%	
Transit disorder	3/15	20%	
Headache	2/15	13%	
Myalgia	1/15	7%	
Arthralgia	1/15	7%	
Dysuria and/or Alguria	7/15	47%	
History of canker sores	10/15	67%	

*Upon clinical examination*: 73% of cases presented multiple ulcerations and 40% had three or more vulvar ulcers. Ulcers were more commonly located on the inner labia and all patients reported the ulcers as painful. In our series, two cases of necrotic ulcers and one of “kissing ulcers” were described.

*Laboratory exams*: the PCR test most performed (see Table 3) was for HSV1 and HSV2 using a vaginal or vulvar smear. This test was conducted in 14 of the 15 patients, including all virgo patients except one. Six patients were also tested for *N. gonorrhoeae* and *C. trachomatis*, three were virgo and three had engaged in sexual intercourse within the three months prior to ulcer onset. We reported a positive result for HSV1 indicating a primo infection of a virgo patient as well as a positive HSV2 test in a patient belonging to Group 1. One patient underwent local sampling for EBV using a vulvar PCR, yielding a negative result. The most requested serology (see Table 4) was for EBV, performed in seven patients: IgG was positive in five patients and only one case had both positive IgG and IgM. CMV was tested in four patients, being positive in one, while PVB19 was tested in one case, being negative. Serology for *T. pallidum* was performed three times in total. HIV1/2 infection was tested in two patients, one from Group 1 and the other from Group 3, but with a maternal history of HIV positivity. Vulvar and/or vaginal bacteriological cultures were performed in seven patients, with six cultures showing normal amounts of physiological flora and one with *S. epidermidis* contamination. Blood counts were performed in nine patients, detecting pathological C-reactive protein levels in seven. Two patients had hyperleukocytosis while thrombocytopenia was found in only three patients.

**Table 3:** PCR for STIs (HSV2, *N. gonorrhoeae*, *C. trachomatis*) and other infections (HSV1, VZV).

	STI			Other infections	
	HSV2	*N. gonorrhoeae*	*C. trachomatis*	HSV1	VZV
PCR performed	14/15	6/15	6/15	14/15	11/15
PCR positive	1/14	0/6	0/15	1/14	0/11
PCR Group 1	4/4	3/4	3/4	4/4	3/4
PCR Group 2	2/2	0/2	0/2	2/2	1/2
PCR Group 3	8/9	3/9	3/9	8/9	7/9

PCR, polymerase chain reaction; NP, not performed; −, Negative; +, Positive; Group 1, sexual intercourse within the previous three months; Group 2, non-virgo patients and not sexually active within the three months before the ulcer lesion; Group 3, virgo patients.

**Table 4:** Serologies for CMV, EBV, *T. pallidum*, and HIV 1/2 infections.

	CMV		EBV		*T. pallidum*	HIV-1/2 + HIV Ag p24
	IgM	IgG	IgM	IgG		
SERO performed	4/15	4/15	7/15	7/15	3/15	3/15
SERO positive	0/4	1/4	1/7	5/7	0/3	0/3
SERO Groupe 1	3/4	3/4	3/4	3/4	2/4	2/4
SERO Groupe 2	0/2	0/2	0/2	0/2	0/2	0/2
SERO Groupe 3	1/9	1/9	4/9	4/9	1/9	1/9

SERO, serology; NP, not performed; −, Negative; +, Positive; =, result not available/Cancelled in progress; Group 1, sexual intercourse within the previous three months; Group 2, non-virgo patients and not sexually active within the three months before the ulcer lesion; Group 3, virgo patients.

*Biopsies*: a diagnostic biopsy with histopathology analysis was performed on two patients. The first case involved a virgo patient presenting a necrotic vulvar ulceration accompanied by a febrile state, where infection or melanoma were suspected. The ulcer biopsy was analyzed and described by dermatopathologists as: “ulcer with abcessive dermatitis and thrombosing vasculitis, likely to be a UVAL”. They excluded a herpetic or cancerogenic origin, without being able to definitively exclude EBV infection, Behcet's, or Crohn's disease. In the second patient, belonging to Groupe 2, a histopathological examination was requested due to the presence of a recurrent vulvar lesion of undetermined origin and repeated negative HSV serology results. The histological diagnosis was an inflamed papillary hidradenoma with no evidence of a herpetic lesion and a suspicion of HPV infection. The final clinical diagnosis was infectious vulvar ulceration of undetermined origin.

*Treatments*: the most prescribed analgesic treatment was the local anesthetic lidocaine (Xylocaine gel®) in 87% of patients, followed by oral paracetamol and oral NSAIDs. Four patients requested opioid analgesic treatment due to severe ulcer pain and five patients received treatment for herpes (acyclovir, valacyclovir). Systemic antibiotic was administered in 4 cases, while topical steroids were prescribed for five patients.

*Ulcer healing/outcome*: in all patients except one, wound healing was objectified; this one patient continued her follow-up with her pediatrician; thus, the evolution of the lesion could not be documented at CHUV'ER. The average follow-up lasted 10 days, (with a maximum of 34 days between the first and last visit, with five visits in total). Three of the fifteen cases had recurrent vulvar ulcers (20% of cases); all three were finally diagnosed with Behçet's disease.

*Diagnostic work-up in the Gyn ER:* in nine patients (60%), a diagnosis of UVAL was referred to in the differential diagnosis, however, UVAL was finally accepted as the most plausible diagnosis only in six of all our cases (40%). Of these six patients, one patient had engaged in protected sex with a stable partner while five patients were virgo. For one virgo patient UVAL was maintained as concomitant to HSV1 infection (Table 5).

**Table 5:** At the Gyn ER: UVAL within differential diagnosis; final UVAL diagnosis; other diagnoses*.*

	DD with UVAL	Final DX of UVAL	Diagnoses	*n*
Group 1	+	+	UVAL	1
	+	−	/	2
	−	−	Behcet's	3
	−	−	HSV2 Infection	4
Group 2	−	−	/	5
	−	−	Behcet's	6
Group 3	+	+	UVAL	7
	+	+	UVAL	8
	+	+	UVAL	9
	+	+	UVAL	10
	+	+	Concomitant UVAL and primary HSV1 infection	11
	−	−	/	12
	+	−	/	13
	+	−	/	14
	−	−	Behcet's	15
Synthesis	9/15	6/15		
Percentage	60%	40%		

/, vulvar ulcer of undetermined origin; DD, differential diagnosis; DX, diagnostic.

*Diagnostic algorithm application:* in our series, the total number of cases diagnosed as UVAL by both the algorithm and CHUV's ER gynecologists where five out of fifteen. Of the fifteen cases, only one case was not diagnosed as UVAL by the algorithm but received a UVAL diagnosis upon evaluation at CHUV's gynecological ER. This case was that in which UVAL was maintained as concomitant to HSV1 infection, thus not conforming to the algorithm's major criterion (exclusion of infectious causes). On the contrary, UVAL was retrospectively diagnosed in five patients by the algorithm but not diagnosed in the gynecological ER. Finally, in four of the fifteen cases, the UVAL diagnosis was not established either by the algorithm or the ER gynecologists. Of these four patients, one presented a positive HSV2 PCR, and the other three had Behcet's disease as the final diagnosis (Table 6).

**Table 6:** Number of cases retrospectively diagnosed as UVAL by the algorithm (presence/absence of criteria) compared with those diagnosed in CHUV's gynecological ER.

	UVAL diagnosis at the Gyn-ER	Non-UVAL diagnosis at Gyn-ER	Total
UVAL Diagnostic algorithm *One 1° criterion and minimum two 2° criteria present*	5/15	5/15	10/15
Non-UVAL diagnostic algorithm *1° and 2° criteria absent*	1/15	4/15	5/15
Total	6/15	9/15	

## Discussion

### UVAL: not only young virgo

Of the fifteen patients included in our study, with an average age of 15 years, nine had not yet engaged in sexual intercourse at the time of vulvar ulcer onset. This shows our sample to be representative as in Switzerland 50% of adolescents become sexually active by the age of 17 (16). Furthermore, among patients who have been retrospectively diagnosed with UVAL by the algorithm: seven were virgo, two had engaged in sexual intercourse within three months prior to ulcer onset and, in one case, the patient was neither virgo nor sexually active. Therefore, to us, it seems reasonable to consider the sexual history criteria of the diagnostic algorithm as secondary rather than as an exclusive criterion, changing the common perception that UVAL is a disease that only affects virgo patients. This awareness could help direct questions related to sexual activity more carefully. It is still relevant to sensitively screen for sexual injury, or abuse, nevertheless specific skills in history-taking are necessary and a deeper knowledge on acute vulvar ulcer clinical features (7), as described in our series, could help clinicians not to insist if UVAL diagnostic criteria are met.

### When to expect it?

In the great majority of UVAL cases described in the literature, vulvar ulcer has been consistently associated with non-gynecological symptoms. Some authors (17, 18) even tried to classify ulcers into different subtypes according to their appearance and associated symptoms: a gangrenous form, usually linked to a flu-like inaugural condition; a miliary form with mild general symptoms; and a chronic form characterized by relapsing ulcers. In our cohort, all patients presented at least one flu-like symptom inaugural to ulcer onset, with most cases reporting high fever or an upper respiratory tract infection concomitant to vulvar lesion appearance; this is in line with results from systematic reviews associating UVAL with either a flu-like or mononucleosis-like syndrome (5). It is true that, in a minority of cases (5, 8), the presence of these symptoms was not described as the sine-qua-non criterion for UVAL diagnosis, thus we consider it appropriate for this criterion to be in the secondary category of the diagnostic algorithm.

In our sample, 73% of cases had multiple ulcerations: most vulvar lesions were anatomically located on the inner labia and a minority of them had another location (para-clitoral, vestibular, outer labia, or peri-anal). There were two reported cases of necrotic ulcer and only one case of “kissing ulcers”. This draws a rather varied picture that does not allow us to target a single evocative element for UVAL lesions. So again, it seems consistent with our data to choose “appearance of a single or multiple ulcers in the vulvar region” as a major criterion, and specific ulcer location “either of the inner lips or at the level of the vestibule” as a minor one.

### Which test to perform for diagnosis?

Based on our data, testing for sexually transmitted diseases (STDs) varied on a case-by-case basis. While *N. gonorrhoeae*, *C. trachomatis*, HIV, and syphilitic infection testing was not systematic, HSV2 and HSV1 were tested in all patients except one. Among the nine patients in the virgo group, eight were tested for both HSV1 and HSV2, resulting in only one positive result for HSV-1.

**Figure 2:**
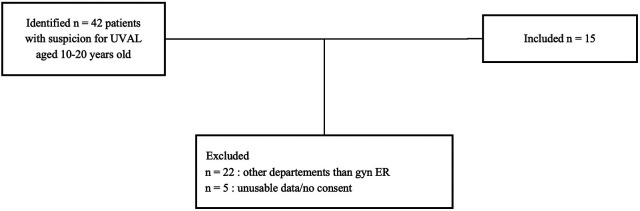
Flow-chart of eligible patients.

**Figure 3:**
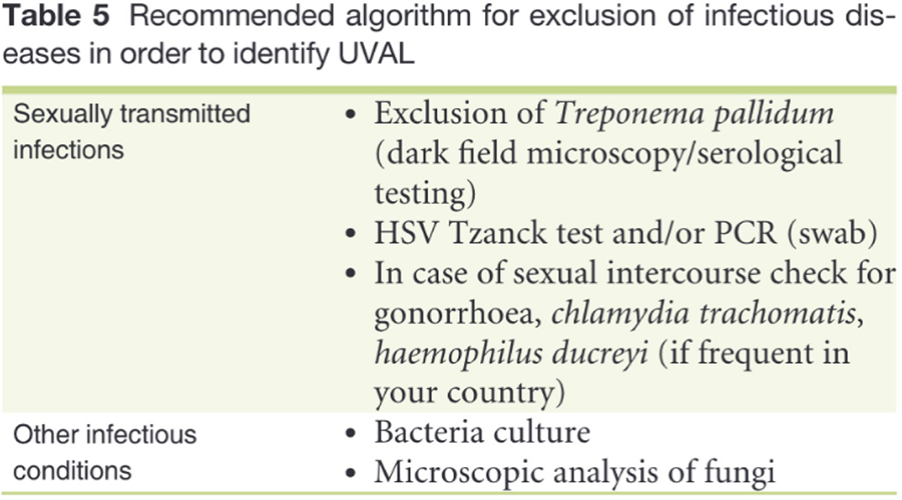
Table 5 of Sadoghi et al. article (8).

Regarding systemic infections, EBV testing was conducted in eight out of fifteen patients due to the presence of mononucleosis-like symptoms and the often-discussed association between UVAL and EBV, although no established etiopathogenic link has been proved (8, 19), as for the association with CMV and PVB19 (10, 20). Only one patient exhibiting both EBV IgG + IgM + serologies, indicating a likely acute phase. Our results suggest that serologies for EBV or CMV can help address the algorithm's fourth minor criterion of systemic infection within 2–4 weeks prior to the onset of vulvar ulcer. However, UVAL diagnosis should not solely rely on their positivity if other criteria are met. As shown in Tables 3 and 4 of our results, the unsystematic and sometimes inconsistent choice of which laboratory test to perform, indicates the need for a more precise diagnostic guideline. When addressing an acute vulvar lesion, we encourage using Sadoghi et al. algorithm with their respective tables (see Figures 2 and 3), as well as to always adapt laboratory examinations to the patient's clinical history.

As mentioned earlier, histological analysis conducted in several studies (3, 11, 21) revealed a non-specific lymphocytic infiltrate in the dermis making biopsies ineffective for UVAL diagnostics. In our series, both histopathological analyses were inconclusive in providing a specific diagnosis. It should be considered that biopsy is highly invasive and that their indication should be greatly discussed (3, 14), particularly in young patients. Fahri et al. suggested that biopsies may be performed in cases with unfavorable progression, when the diagnosis remains elusive even after one month of non-healing, or when there is a high suspicion of vulvar intraepithelial neoplasia (21).

### How to treat?

The most prescribed treatments in our cohort were: oral NSAIDs, Paracetamol, and lidocaine gel to be applied on the vulvar lesion. During clinical examination, all ulcers were described as painful, but 27% of cases presented severe pain and required opioid analgesic treatment. Our treatment data is consistent with the suggested division between painful UVAL, highly inflamed UVAL, and high inflammatory parameters +/− febrile state, proposed by the therapeutic algorithm of Sadoghi et al., which directs a more precise treatment (8). Three patients were initially treated for herpetic infection while awaiting their HSV1 and HSV2 PCR results, which came back negative. Two were diagnosed with UVAL by the algorithm, while the third finally received a Behcet's diagnosis. One patient (patient no. 11 see Table 5) was positive for an HSV1 primo infection; however, acyclovir treatment was described as ineffective, and the ulcer started to heal only when betamethasone was introduced. Clinicians, therefore, made a diagnosis of herpetic primo-infection concomitant to UVAL, rejected by the diagnostic algorithm's second major criterion. This data indirectly shows the need for both more appropriate diagnostic and therapeutic guidelines.

UVAL healing is spontaneous (11) but can take from few days to weeks. We observed an average time for ulcer management of 10 days, with ulcer resolution observed at follow-up in fourteen patients (follow-up lost in one case).

### An algorithm at your fingertips

The diagnoses made in CHUV's gynecological ER were as follows: six cases of UVAL, five patients for whom a precise diagnosis could not be given (we commonly find in the files “vulvar ulcer of undetermined or X origin”,) as well as one patient diagnosed with HSV2 infection, and three patients ultimately diagnosed with Behcet's disease. It's interesting to note that Behcet disease was only considered in the case of relapsing ulcers, i.e., when a long-term follow-up had been initiated because of the recurrence. Vulvar ulcer may in fact be the first symptom of this disease, something which is still considered rare in Western Europe but that often affecting young patients (22, 23) and, therefore, likely to be diagnosed in a population comparable to ours. A second frequently overlapping element between UVAL and Behcet's is the positive history of oral canker sores. In our sample, we found a history of recurrent oral canker sores in 10 out of 15 patients and we believe that this should be systematically investigated when gathering the medical history (13). Oral aphthae are present in several diagnoses such as Behcet's disease, Crohn's disease, and idiopathic recurrent aphthosis [a diagnosis requiring recurrence of both vulvar and genital aphthae (24)], but according to our findings, we could argue that the presence of oral ulcer is not exclusive to these diagnoses. Hence, when it is present, a diagnosis of UVAL still has its legitimacy (25).

Finally, considering the retrospective application of the diagnostic algorithm on our data allowed us to assign a UVAL diagnosis to five previously undiagnosed patients, reject a UVAL misdiagnosis in one case (concomitant HSV1 infection) and confirm four cases as non-UVAL. This indirectly evidences that, when it comes to diseases other than UVAL, clinicians may recognize and thus diagnose more promptly and that there is a veritable need to include UVAL within our clinical differential diagnostic reasoning.

**Figure 4:**
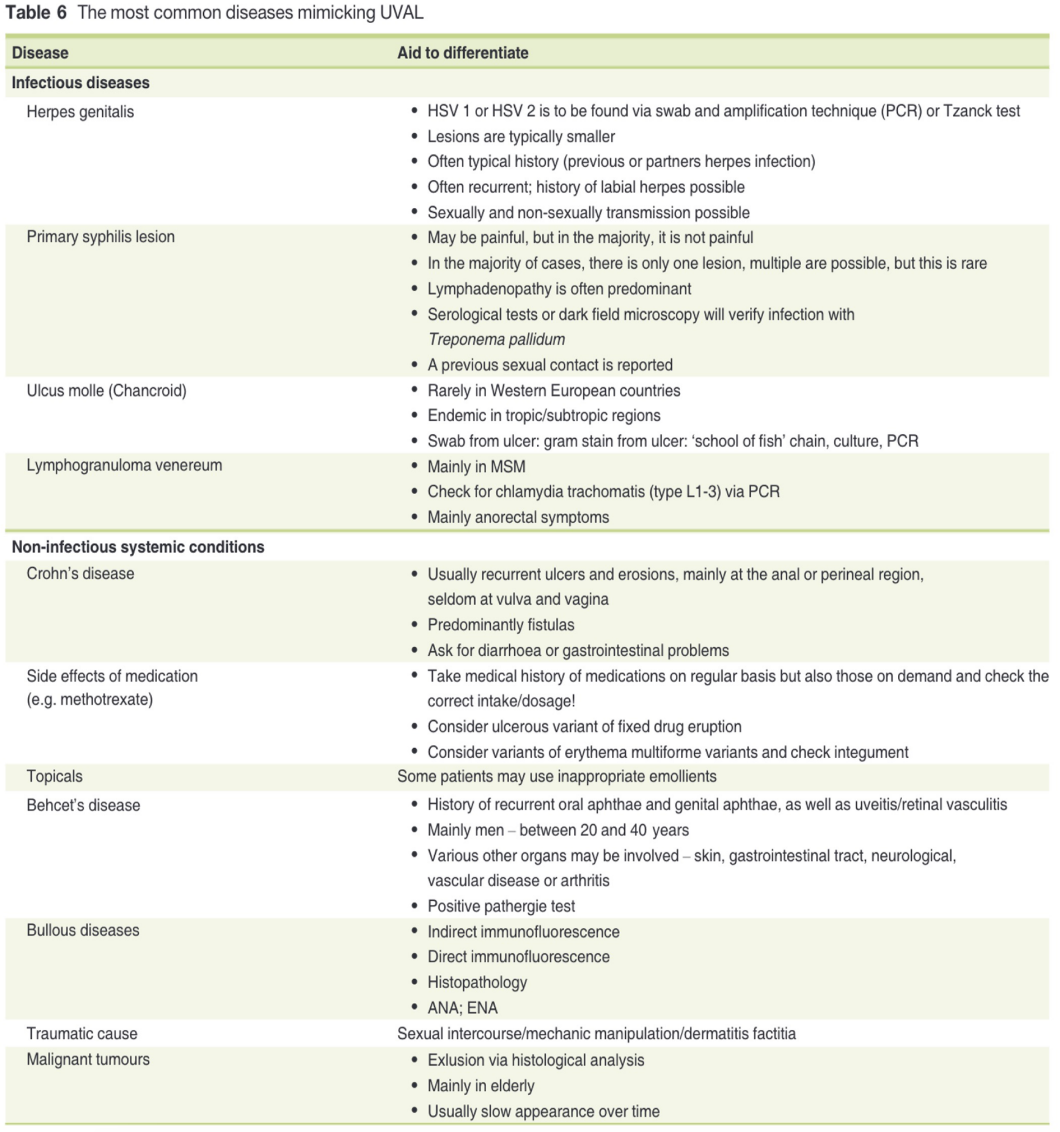
Table 6 of Sadoghi et al. article (8).

### Limitations

Our study was limited primarily by its retrospective nature: UVAL incidence remains unknown to date, and we lacked data concerning follow-up and treatment effectiveness. We were only able to collect data on the ongoing regression of vulvar ulcers but not their definitive appropriate or inappropriate healing (we were able to make an indirect estimate of UVAL duration measured in days between the first and last check-up and calculating an imperfect indirect endpoint). We have no data concerning the sequelae that these ulcerations may have caused both on the symptomatologic and psychological level in our young patients. Some records were scanned from handwritten, illegible forms, contributing to data loss for older records. Additionally, it is noteworthy that even though several cases of UVAL have been described in the pediatric population, we had to exclude 22 patients who were identified with a suspicion of UVAL. Among these patients, 10 were pediatric patients, and they were excluded based on our age criteria. However, we believe that the good diagnostic and therapeutic practices analyzed in our study can be equally beneficial for pediatricians who encounter UVAL disease, considering its rare and relatively unknown nature.

## Conclusion

Our case series highlights the difficulties related to UVAL diagnosis. The disease often affects virgo patients but not exclusively. Clinical information needs to be gathered considering the diverse manifestations of acute vulvar ulcers: UVAL diagnosis remains one of exclusion with a wide and complex range of conditions falling within its differential diagnosis. This can sometimes lead to excessive investigations and practitioners should choose suitable exams only in the presence of evocative anamnestic elements: we consider some, such as serologies and PCRs, more appropriate than others, as for biopsies with histopathological analysis. As previously discussed, we recommend all clinicians to use jointly Sadoghi et al. (8) diagnostic and therapeutic algorithms, thus implementing UVAL management, which nowadays still requires a more precise diagnostic approach.

## Data Availability

The data analyzed in this study is subject to the following licenses/restrictions: The dataset used for analysis is protected by CHUV's university hospital data system. Requests to access these datasets should be directed to https://www.chuv.ch/fr/fiches/direction-des-systemes-dinformation.

## References

[1] Lipschütz B. Über eine eigenartige Geschwürsform des weiblichen Genitales (Ulcus vulvae acutum). Arch Dermatol Syph. (1913) 114(1):363–96. 10.1007/BF01973166

[2] Kluger N Garcia C Guillot B. Acute ulcer of Lipschütz’s vulva. J Gynecol Obstet Biol Reprod. (2009) 38(6):528–30. 10.1016/j.jgyn.2009.08.005 19744804

[3] Lehman JS Bruce AJ Wetter DA Ferguson SB Rogers RS. Reactive nonsexually related acute genital ulcers: review of cases evaluated at Mayo clinic. J Am Acad Dermatol. (2010) 63(1):44–51. 10.1016/j.jaad.2009.08.038 20462661

[4] Chen W Plewig G. Lipschutz genital ulcer revisited: is juvenile gangrenous vasculitis of the scrotum the male counterpart. J Eur Acad Dermatol Venereol. (2019) 33:1660–6. 10.1111/jdv.15598 30903712

[5] Vismara SA Lava SAG Kottanattu L Simonetti GD Zgraggen L Clericetti CM Lipschütz’s acute vulvar ulcer: a systematic review. Eur J Pediatr. (2020) 179:1559–67. 10.1007/s00431-020-03647-y 32296983

[6] Leal AA S Piccinato CA Beck APA Gomes MTV Podgaec S. Acute genital ulcers: keep Lipschütz ulcer in mind. Arch Gynecol Obstet. (2018) 298(5):927–31. 10.1007/s00404-018-4866-6 30143859

[7] García JG Pavón BM Martín LM Martínez BF Norniella CM Caro FÁ. Lipschütz ulcer: a cause of misdiagnosis when suspecting child abuse. Am J Emerg Med. (2016) 34(7):1326.e1–e2. 10.1016/j.ajem.2015.12.027 26782798

[8] Sadoghi B Stary G Wolf P Komericki P. Ulcus vulvae acutum Lipschütz: a systematic literature review and a diagnostic and therapeutic algorithm. J Eur Acad Dermatol Venereol. (2020) 34(7):1432–9. 10.1111/jdv.16161 31855308 PMC7496640

[9] Delgado-Garcia S Palacios-Marques A Martinez-Escoriza JC Martin-Bayon TA. Acute genital ulcers. Case Reports (2014).

[10] Vieira-Baptista P Lima-Silva J Beires J Martinez-de-Oliveira J. Lipschütz ulcers: should we rethink this? An analysis of 33 cases. Eur J Obstet Gynecol Reprod Biol. (2016) 198:149–52. 10.1016/j.ejogrb.2015.07.016 26297242

[11] Govindan B. Lipschütz ulcers: a Literature review based on 79 cases. EMJ Repro Health. (2016) 2(1):73–8. 10.33590/emjreprohealth/10311698

[12] Austin AL Magana JN Rudinsky SL Portouw SJ. Case report: not all genital ulcers are herpetic. Pediatr Emerg Care. (2018) 34(4):E73–4. 10.1097/PEC.0000000000000761 27331577

[13] Huppert JS Gerber MA Deitch HR Mortensen JE Staat MA Adams Hillard PJ. Vulvar ulcers in young females: a manifestation of aphthosis. J Pediatr Adolesc Gynecol. (2006) 19(13):195–204. 10.1016/j.jpag.2006.02.006 16731413

[14] Moise A Nervo P Dean J Kridelka F Maquet J Vandenbossche G. Ulcer of Lipschutz, a rare and unknown cause of genital ulceration. Facts Views Vis Obgyn. (2018) 10(1):55–7. PMID: 30510669 30510669 PMC6260674

[15] International Classification of Diseases. ICD-11 for Mortality and Morbidity Statistics codes: GA00.3, GA13.Y GA13.Z, GA1Y, GA1Z. Available at: https://icd.who.int/browse11/l-m/en (Updated 01/2023) (Cited June 2023).

[16] Federal Commission for Children and Young People CFEJ—Federal Department of Home Affairs FDHA. Young people’s sexuality over time: evolution, influences and perspectives, Bern (2009).

[17] Török L Domján K Faragó E Kecskemét H. Ulcus vulvae acutum. Cutis. (2000) 65(6):387–9. 10879308

[18] Plácido Paias R Portillo Márquez M Castillo Navio E González Carracedo MJ Vaquerizo V. Lipschütz ulcer: gangrenous form. Rev Pediatr Aten primaria. (2014) 16(64):e151–4. 10.4321/S1139-76322014000500013

[19] Nicolas X Ansart S Jaffuel S Deluc A Le Berre R Tandé D Genital ulcerations during primary Epstein-Barr virus infection. J Intern Med. (2005) 26(11):913–6. 10.1016/j.revmed.2005.07.008 16129521

[20] Huppert JS. Lipschutz ulcers: evaluation and management of acute genital ulcers in women: Lipschutz ulcers. Dermatol Ther. (2010) 23(5):533–40. 10.1111/j.1529-8019.2010.01356.x 20868407

[21] Farhi D Wendling J Molinari E Raynal J Carcelain G Morand P Non-sexually related acute genital ulcers in 13 pubertal girls: a clinical and microbiological study. Arch Dermatol. (2009) 145:38–45. 10.1001/archdermatol.2008.519 19153341

[22] Masmejan S Guex-Crosier Y Diserens C Vouga M Clottu AS Ribi C When obstetrics–gynecology specialists need to call an ophthalmologist urgently: a case report. J Med Case Rep. (2021) 15(1):517. 10.1186/s13256-021-03087-8 34670612 PMC8529822

[23] Alkazzaz AMH Ebdan WR Ghoben MK Kareem ZT Al-Harbi SJO. Behcet’s disease in Iraq: new insights into the clinical and epidemiologic features in Middle-Euphrates region. Expert Rev Clin Immunol. (2020) 16(1):109–12. 10.1080/1744666X.2019.1705786 31851853

[24] Martín JM Godoy R Calduch L Villalon G Jordá E. Lipschütz acute vulval ulcers associated with primary cytomegalovirus infection. Pediatr Dermatol. (2008) 25(1):113–5. 10.1111/j.1525-1470.2007.00597.x 18304169

[25] Rogers RS III. Recurrent aphthous stomatitis: clinical characteristics and associated systemic disorders. Semin Cutan Med Surg. (1997) 16:278–83. 10.1016/S1085-5629(97)80017-X 9421219

